# Emodin inhibits bladder inflammation and fibrosis in mice with interstitial cystitis by regulating JMJD3

**DOI:** 10.1590/acb385123

**Published:** 2023-12-01

**Authors:** Junyu Lai, Xing Liu, Hongwei Su, Yongsheng Zhu, Ke Xin, Mingwei Huang, Songtao Luo, Hai Tang

**Affiliations:** 1 Southwest Medical University – Affiliated TCM Hospital – Department of Urology – Luzhou (Sichuan) – China.; 2 Southwest Medical University – Affiliated Hospital – Department of Urology – Luzhou (Sichuan) – China.

**Keywords:** Cystitis, Interstitial, Emodin, Inflammation, Fibrosis

## Abstract

**Purpose:**

Interstitial cystitis/bladder pain syndrome (IC/BPS) is a devastating urological chronic pelvic pain condition. In search of a potential treatment, we investigated the effect of emodin on IC/BPS inflammation and fibrosis, and explore the potential mechanism.

**Methods:**

An experimental model of interstitial cystitis was induced by cyclophosphamide, and human bladder smooth muscle cells were treated with lipopolysaccharide to establish the cell model *in vitro*. In both models, inflammation- and fibrosis-related indexes were measured after emodin administration. Furthermore, the specific antagonists were used to dig for the mechanisms underlying the response to emodin treatment.

**Results:**

Emodin significantly ameliorated management of cystitis, reduced the amount of inflammatory cytokines (tumor necrosis factor-α, monocyte chemoattractant protein-1, interleukin-1β, interleukin-8, and interleukin-6) in models, as well as reducing the synthesis of fibrosis marker including collagen1, collagen3, vimentin, fibronectin and α-smooth muscle actin. Further mechanism studies demonstrated that emodin inhibited inflammatory reaction and fibrosis through blocking lysine-specific demethylase 6B (JMJD3) expression via JAK/STAT, NF-κB and TGF-β/SMAD pathways.

**Conclusions:**

Our study reveals the critical role of emodin-JMJD3 signaling in interstitial cystitis by regulating inflammation, fibrosis, and extracellular matrix deposition in cells and tissues, and these findings provide an avenue for effective treatment of patients with cystitis.

## Introduction

Interstitial cystitis/bladder pain syndrome (IC/BPS) is a chronic disease characterized by incapacitating pelvic pain^1^. Especially in Western countries, currently numbers ~6,530 females and ~4,200 males per 100,000 persons diagnosed with IC^2^. Pentosan polysulphate sodium (PPS), a U.S. Food and Drug Administration approved medication for IC^3^, was listed as a Class 2B carcinogen in the preliminary reference to the list of carcinogens published by the World Health Organization’s International Agency for Research on Cancer in 2017^4^. Thus, it is imminent to find new effective drugs for IC with less toxicity.

While the precise etiology of IC remains unclear, increasing evidence supports the roles of immune modulation in exacerbating IC^5^. In IC patients, the main histological finding is chronic inflammation in bladder tissues, and bladder-centric symptoms are predominant^6^. Furthermore, an inappropriate chronic inflammatory response occurs, resulting in progressive fibrosis with an excessive accumulation of extracellular matrix (ECM) components^7^, and fibrosis in the bladder of IC/BPS patients was correlated with increased urinary frequency and decreased bladder capacity^8^.

Therefore, an effective treatment to block the inflammation process in bladder tissues is a potential innovation therapy for IC^3^. Lysine-specific demethylase 6B (JMJD3, also known as KDM6B) is a demethylase of H3K27me3. Previous studies demonstrated that JMJD3 is highly expressed in inflammation^9^. Chen et al. found that JMJD3 promoted the expression of interleukin (IL)-1β in early sepsis^10^. In addition JMJD3 can regulate the proliferation of neointima following vascular injury^11^, and promote joint destruction in rheumatoid arthritis by boosting the proliferation and migration of fibroblast-like synoviocyte^12^. In the urinary system, JMJD3 was postulated to be associated with the etiology of IC/BPS^13^. Hence, JMJD3, an epigenetic regulator, may have crucial effects on pathological and physiological activities of bladder reconstruction.

Emodin (1,3,8-trihydroxy-6-methylanthraquinone), a natural anthraquinone derivative contained in *Rheum ribes* L., is mainly used in traditional Chinese medicine to treat sore throats, carbuncles, sores, blood stasis, and damp-heat jaundice^14^. Emodin pharmacological research in the last decade has revealed other potential therapeutic applications such as anticancer, neuroprotective, antidiabetic, anti-oxidation, anti-inflammatory and anti-fibrotic by regulating multiple signaling pathways^14^^–^ 16. For example, emodin has been shown to have therapeutic effects on many human malignant tumors, such as lung carcinoma, hepatoma, leukemia, and cervical cancer^17^^–^ 19.

One of its mechanisms of action is to inhibit the biosynthesis of DNA, RNA and protein in cancer cells and inhibit the oxidative dehydrogenation of cancer cells. In addition, emodin exert anti-inflammatory effects by blocking mitogen-activated protein kinase (MAPK) and PI3K/Akt pathway signaling^20^^,^ 21 and antioxidation effects by inhibiting the activation of nuclear factor kappa-B (NF-κB) and inductible nitric oxide synthase (iNOS) expression^22^. Hu et al. demonstrated that emodin confers an anti-inflammatory effect by suppressing the production of nitric oxide (NO), IL-6, and IL-1β in lipopolysaccharide (LPS)-induced RAW264.7 macrophages via inhibition of NF-κB, MAPK, and PI3K pathways^23^. Currently, the development of medicinal plants and the research on the mechanism of natural active products are still research hotspots, especially those related to immune checkpoints^24^.

However, the effect of emodin on inflammation-induced bladder injury has not been studied. We hypothesized that the downregulation of JMJD3 expression in bladder tissues by emodin can attenuate bladder injury in IC. Thus, we evaluated the therapeutic effect of emodin and its possible therapeutic mechanisms in inflammation-induced bladder injury disease by using a bladder-centric mouse model.

## Methods

### The in-vivo model and related methods

#### Experimental animals

This study was approved by the Ethics Committee of Southwest Medical University Laboratory Animal Welfare. All experimental procedures in mice were conformed with the Guide for the Care and Use of Laboratory Animals of the National Institutes of Health. Female C57BL/6 mice (aged 7–9 weeks old, weighing 18–22 g) were housed with normal diet in a cycle of 12-h light and 12-h dark for a week of acclimatization. All possible efforts were made to reduce animal pain and stress.

#### Interstitial cystitis mouse model and interventions

Dimethyl sulfoxide (DMSO) was purchased from Merck (Darmstadt, Germany). The SB431542 working solution (1μM) was freshly prepared with 0.1% DMSO. We administered cyclophosphamide (CYP) (200 mg/kg) to mice by intraperitoneal injection to induce IC damage. Concretely, cystitis mouse model was induced by intraperitoneally injection of 200 mg/200 μL/1 kg of cyclophosphamide (CYP; ICN Biomedicals, Aurora, OH) on alternate days (Day 1, 3, and 5)^25^^,^ 26, while mice in the control (ctrl) group was injected with phosphate buffer solution (PBS).

The interventions were carried out in two phases: PBS, Emodin (10 mg/kg), GSK-J4 (JMJD3 inhibitor, SML0701, Sigma, United States of America; 10 mg/kg), and SB431542 (ALK5/TGF-β1 inhibitor, HY-10431, MCE, United States of America; 100 μL/mice) were given intraperitoneally to mice, and JSH23 (NF-κB inhibitor, ab144824, Abcam, USA; 3 mg/kg) was gavaged to mice at 0.5 h prior to CYP exposure, respectively. Then, the same intervention procedures were followed for another two weeks after CYP injection. Mice were sacrificed at experimental end point following CYP/reagents treatment, and the bladder and blood samples were collected for future analysis.

#### Histological analysis

The bladder histomorphology was examined via hematoxylin-eosin (HE) staining. Briefly, the excised bladder was fixed in 4% paraformaldehyde. The specimens were excised, and paraffin sections of 4–6 μm thickness were created and then mounted on glass slides for HE staining (G1120, Sole Bauer) according to standard protocols. Sections were examined under an inverted microscope (DMIL-PH1, Leica, German).

#### Immunofluorescence staining of mouse bladder

Immunofluorescence staining was performed to visualize JMJD3 in bladder. Briefly, mouse bladders were fixed in 4% paraformaldehyde at 4°C overnight, and then dehydrated sequentially in 10–30% of sucrose solution each at 4°C. The samples were embedded into the optimal cutting temperature compound (Sakura Finetek, Torrance, CA, United States of America), and 5-μ-thick sections were permeabilized and simultaneously blocked with 0.5% Triton X-100 and 5% goat serum. The sections were incubated with primary antibodies against JMJD3 (1:100 diluted, cat: orb75718, Biorbyt, Cambridge, United Kingdom) at 4°C overnight, and further incubated with secondary antibodies–goat anti-Rabbit IgG (H+L) Highly Cross-Adsorbed Secondary Antibody (Alexa Fluor™ 488; cat: A-11034, Thermo Fisher, Massachusetts, United States of America)–at room temperature (RT) for 1 hour in the dark. Finally, slides were photographed using a fluorescence microscope. The intensities of JMJD3 (green fluorescence) were obtained using Image J software (National Institutes of Health, Bethesda, MD, United States of America) and normalized against 4’,6-diamidino-2-phenylindole (DAPI) intensities.

#### Masson stain

A Masson’s trichrome kit (Masson’s Trichrome Stain Kit, G1340, Solarbio, China) was used to detect collagen fibers in the urinary bladders of IC-mice. First, dehydrated frozen slides were stained with a working solution of Weigert’s iron hematoxylin for 5 min. Then, they were incubated with an acid fuchsin solution for 15 min and rinsed in distilled water. Subsequently, the slides were developed in phosphomolybdic/phosphotungstic acid solution for 15 min until the collagen fibers became discolored. Afterwards, aniline blue solution was directly applied to the slides for 10 min. The slides were again rinsed and treated with 1% acetic acid solution for 5 min, dehydrated with 95% alcohol twice, and washed with absolute alcohol. The slides were cleared in xylene and mounted using a synthetic resin. The bladder sections of each specimen were captured by digital camera, non-overlapping frames at 400X magnification and compared between experimental groups. The color setting and image-associated quantification were determined by image analysis software (Image-Pro Plus, Media Cybernetics, MD, United States of America).

#### Immunohistochemistry

To evaluate the expression of fibrosis-related markers in bladder tissue, formalin-fixed paraffin embedded tissue sections were stained with anti-E-cadherin (#49398, CST, USA), anti-collagen1 (ab34710, Abcam, United States of America), anti-collagen3 (ab7778, Abcam, United States of America), anti-Fibronectin (10314-R014, Sino Biological, China), anti-Vimentin (#49398, CST, United States of America) antibodies at 4°C overnight in a humidified chamber. Horseradish peroxidase (ImmPRESS HRP) polymer kit was applied the next day for 1 hour at RT for secondary antibody incubation. Slides were then incubated with 3,3’-diaminobenzidine (Vector Laboratories) and counterstained with hematoxylin. The mentioned proteins, as markers of ECM, are usually used to judge the rate of fibrosis. For each film, positively stained protein was identified and quantified at 200-fold and 400-fold under microscope. The results were calculated as the content of fiber marker protein per bladder.

### The in-vitro model and related methods

#### Cell culture

Human bladder smooth muscle cells (hBSMCs) were obtained from ScienCell Research Laboratories (Carlsbad, United States of America) and cultured in Dulbecco’s Modified Eagle Medium (DMEM) supplemented with 10% fetal bovine serum (FBS; Gibco) and 1% penicillin–streptomycin (100 U/mL penicillin, and 100 μg/mL streptomycin) in a 5% CO_2_ atmosphere at 37°C. The culture medium was changed every two days.

#### Cell treatment and grouping

hBSMCs were stimulated *in vitro* with LPS (L2880, Sigma, United States of America), a classical reagent for induction of inflammation, commonly used to induce IC in cells or animal subjects to promote the progress of fibrosis^27^. Briefly, hBSMCs were cultured into six-well plates at a density of 1×10^5^ cells/well for 24 hours and categorized into five groups. Next, cells in the control group were not treated with drugs, those in the LPS group were stimulated with LPS (10 μg/mL) for 24 hours, and those in the remaining groups were pretreated with emodin (2.5 μm), eukaryotic plasmid pcDNA3.1-JMJD3 and short hairpin RNA (shRNA JMJD3; Santa Cruz Biotechnology, United States of America), followed by LPS for 24 hours.

#### CCK-8 proliferation assay

The effect of Emodin, pcDNA3.1-JMJD3 and shRNA JMJD3 on the proliferation of LPS-induced hBSMCs was assessed using a CCK-8 kit from Dojindo Molecular Technologies (Rockville, MD, United States of America). hBSMCs were seeded in 96-well plates at a density of 1×10^4^ cells/well for 24 hours with a complete culture medium, grouped and pretreated as described before. Then, cells were incubated with CCK-8 solution at 37°C for 30 min, and the absorbance was detected using a microplate reader (Shanghai Utrao Medical Instrument Co., Ltd., Shanghai, China), according to the manufacturer’s methods. A growth curve was plotted as per the absorbance value.

### The methods used both with the *in-vivo* samples and the in-vitro samples

#### Enzyme linked immunosorbent assay

Tumor necrosis factor (TNF)-α, IL-1β, monocyte chemoattractant protein-1 (MCP-1), IL-6 and IL-8 were detected in serum or hBSMCs by enzyme linked immunosorbent assay (ELISA). For *in-vivo* samples, obtained blood were centrifuged at 1,000 × g at 4°C for 10 minutes. Separated sera were stored at -80°C. For *in-vitro* samples, hBSMCs were seeded in six-well plates with a complete culture medium, incubated to reach 90% confluency followed starving for 12 hours. Cells were grouped and pretreated as described before, and the culture medium was then collected. Concentrations of IL-6, IL-8, IL-1β, MCP-1 and TNF-α were measured using the ELISA kits (Shanghai Xitang Biotechnology Co., Ltd, Shanghai, China) according to the manufacturer’s protocol.

#### RNA isolation and RT-qPCR

Total RNA was extracted from bladder tissue or hBSMCs using a RNeasy Mini Kit (Qiagen, German). RNA concentration was measured using a spectrophotometer (IMPLEN Nanophotometer, Germany). The cDNA was synthesized using RevertAid First Strand cDNA Synthesis Kit (Thermo Scientific). Real-time quantitative polymerase chain reaction (RT-qPCR) was performed using Bio-Rad CFX Manager™ software version 3.1 (Bio-Rad CFX96 instrument)^28^. The forward and reverse primer sequences for JMJD3 were GCCTAAGTTGAGCCGAAGTG and CCCCAGCATCATTTTGGATG, respectively. β-actin was set as an internal control. The PCR reaction was performed using Brilliant II SYBR Green qPCR Master Mix kit (Agilent Technologies, Santa Clara, CA, United States of America). The real-time data were analyzed using the 2^-ΔΔCt^ method. Data were represented as the mean ± standard deviation, and the experiments were performed in triplicate.

#### Western blot analysis

Total protein content was extracted from the tissues and cells by RIPA lysate (P0013B, Beyotime, China) containing protease inhibitor (A8260, Solarbio, China). Next, the obtained proteins underwent SDS-PAGE separation, and electro-transferring onto a poly (vinylidene fluoride) membrane (PVDF; 0.22 μm, Merck, Millipore). Then, 5% skimmed milk was used to block the membranes for 2 hours at room temperature, and further incubated with primary antibodies at 4°C overnight. The membranes were incubated with secondary anti-rabbit IgG or anti-mouse IgG (MBL, Japan) for 1 hour at 37°C. Primary antibodies against JMJD3 (ab169197, Abcam, United States of America), collagen1 (ab34710, Abcam, United States of America), collagen3 (ab7778, Abcam, United States of America), fibronectin (10314-R014, Sino Biological, China), α-SMA (PL0401304, PL Laboratories, Canada), p65 NF-κB (250,060, Zen Bioscience, China), phospho (p)-p65 NF-κB (310,013, phospho Ser536, Zen Bioscience, China), IκBα (07-1483, Merck, Millipore), p-IκBα (Ylk-KT2913D, Ylkbio), TGF-β1 (5559-30T, BioVision, United States of America), SMAD2 (50727-T58, Sino Biological, China), p-SMAD2 (IC195026, Abcam, United States of America), SMAD3 (100424-T36, Sino Biological, China), p-SMAD3 (IC187103, Abcam, United States of America), JAK2 (FNab04432, FineTest, China), p-JAK2 (IC165485, Abcam, United States of America), STAT3 (RHE27701, AntibodySystem, France), p-STAT3 (IC184614, Abcam, United States of America), β-actin (BM0627, Booster, United States of America). Development of membrane was performed with enhanced chemiluminescence (ECL, GE Healthcare) by using a ChemiDoc XRS with Image Lab Software to visualize and measure the intensity of the immunoreactive bands (Bio-Rad Laboratories).

### Statistics

All the experiments were repeated three times, and the results were provided as mean ± standard deviation. Prism statistical computer program (GraphPad 8.0 software, United States of America) was applied in the statistical analysis. One-way analysis of variance was applied to compare the quantitative data from multiple groups, and statistical significance was considered when *P* < 0.05.

## Results

### Emodin inhibits the expression of JMJD3 in interstitial cystitis

**Figure 1:**
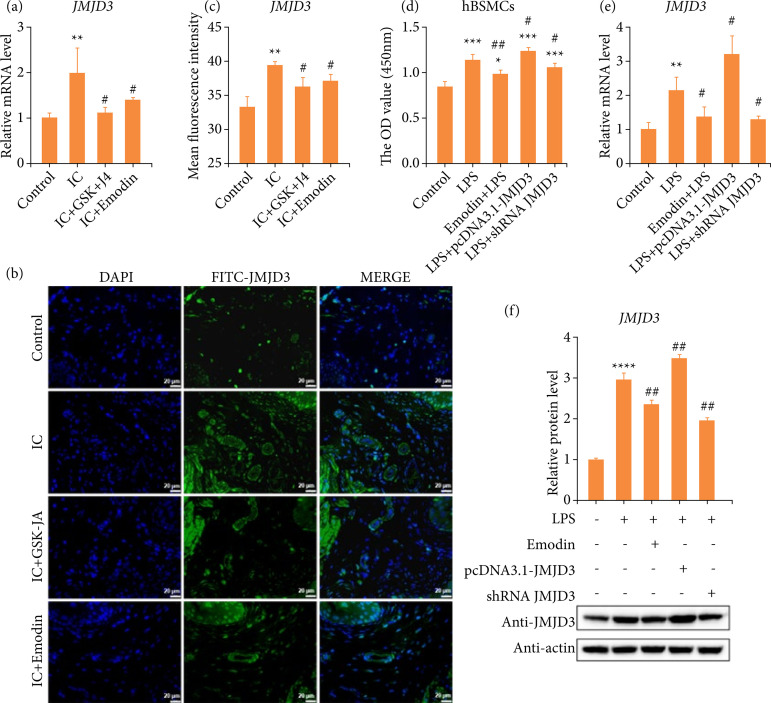
CYP-induced upregulation of JMJD3 expression is significantly reversed by emodin, *in vitro* and *in vivo*. **(a)** The mouse bladders were harvested at the end of the experiment, comparison of gene expression of JMJD3 among all groups, by RT-qPCR. **(b)** JMJD3 expression was analyzed using immunofluorescence. Scale bar = 20 μm final magnification. **(c)** Quantitative analysis of immunofluorescence data. **(d)** hBSMC cell viability was assessed by CCK-8 assay, and the cell viability in intervention groups was not lower than that in control group. **(e)** Comparison of JMJD3 expression in cell samples among all groups, by RT-qPCR. **(f)** Western blot analysis and quantification of JMJD3 protein expression in different groups. Data are represented as the mean ± standard deviation.

Source: Elaborated by the authors. JMJD3: lysine-specific demethylase 6B; IC: interstitial cystitis; OD: optical density; hBSMC: human bladder smooth muscle cells; LPS: lipopolysaccharide; CYP: cyclophosphamide; RT-qPCR: real-time quantitative polymerase chain reaction; compared with control (black rectangle box), * *P* < 0.05, ** *P* < 0.01, *** *P* < 0.001, **** *P* < 0.0001; compared with IC group, **#** *P* < 0.05, **##** *P* < 0.01 represent significant differences between the indicated columns.

To elucidate whether emodin could be used as a potential drug for IC, we have established a mice model of IC by intraperitoneal injection of CYP. Significantly elevated JMJD3 mRNA levels were observed in CYP-stimulated bladders (Fig. 1a). However, this abnormal increase could be reversed by GSK-J4 or the natural ingredient emodin (Fig. 1a). GSK-J4 is a small-molecule inhibitor functions on H3K27 histone demethylase JMJD3/UTX to suppress enzyme activity^29^^,^ 30. Subsequently, quantify immunofluorescence staining results also proved this phenomenon again (Figs. 1b and 1c).

Since we confirmed the regulation of JMJD3 by emodin treatment, we further determined to detect whether JMJD3 was involved in the responses of hBSMCs to external stimuli. An inflamed-hBSMCs model *in vitro* with LPS was used in our study, and CCK8 assay was performed to evaluate the potential toxicity of LPS, emodin, pcDNA3.1-JNJD3 and shRNA JMJD3. The results suggested that the concentration-worked of LPS has positive impact on hBSMCs, and the activity of cells treated with emodin, pcDNA3.1-JNJD3 and shRNA JMJD3 was not lower than that of control (Fig. 1d). Based on the result, our data illustrated emodin and shRNA JMJD3 significantly inhibited JMJD3 transcription (Fig. 1e) and translation (Fig. 1f). As previously mentioned, emodin was able to significantly inhibit the production of JMJD3, both *in vitro* and *in vivo*, as same as available specific inhibitors.

### Emodin inhibits bladder inflammation in mice with interstitial cystitis

**Figure 2:**
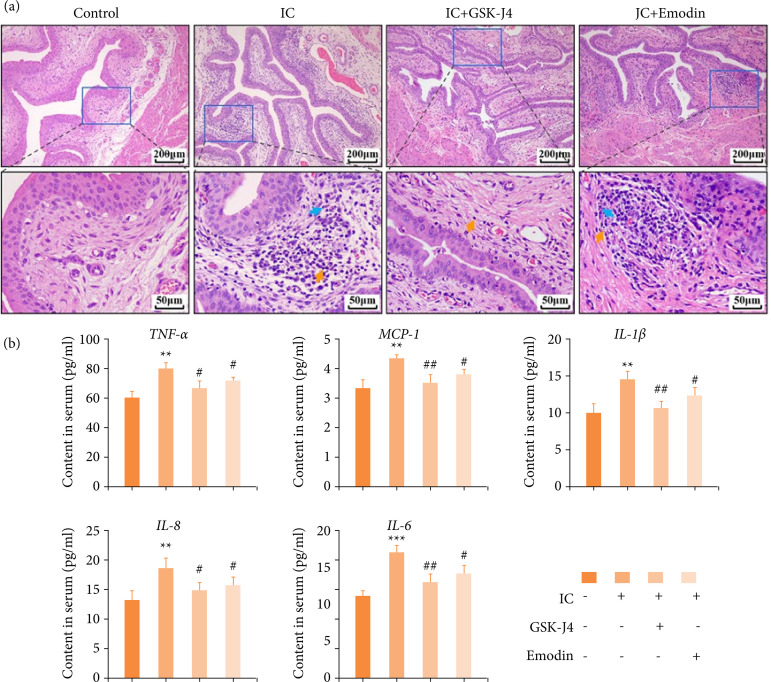
Changes in urinary bladder histology and inflammation after emodin treatment during CYP-induced IC. **(a)** Histomorphology in four groups of mice urinary bladders was analyzed by hematoxylin and eosin staining. 100× (scale bar = 200 μm) and 400× (scale bar = 50 μm) final magnification. **(b)** The content of inflammation-related genes (TNF-α, MCP-1, IL-1β, IL-8, and IL-6) in serum, detected by enzyme-linked immunosorbent assay. Data are represented as the mean ± standard deviation.

Source: Elaborated by the authors. IC: interstitial cystitis; TNF: tumor necrosis factor; MCP: monocyte chemoattractant protein; IL: interleukin; blue arrow: neutrophils; yellow arrow: lymphocytes; compared with control, **P < 0.01, ***P < 0.001; compared with IC group, ^#^ *P* < 0.05, ^##^ *P* < 0.01 represent significant differences between the indicated columns.

Urinary bladders were observed histologically by H&E staining at a high magnification, and the bladder histology was intact, with no visible lesions and inflammatory infiltration in the control group. For IC group, lamina propria was infiltrated with inflammatory cells, including lymphocytes and neutrophils, with mild vascular congestion and accumulation of red blood cells. Otherwise, treatment with emodin or GSK-J4 attenuated bladder damage and inflammation, although its lamina propria still infiltrated with inflammatory cells and erythrocyte (Fig. 2a). Dysregulation of the inflammatory response with excessive release of inflammation mediators is the hallmark of IC. Urinary chemokines and cytokines are considered to be the noninvasive predictor of IC^31^. Levels of cytokines and chemokines were quantified by a Bio-Plex system in the serum from the four groups of mice described before. Significantly elevated serum TNF-α, MCP-1, IL-1β, IL-8 and IL-6 were observed in IC mice compared to control mice (Fig. 2b). Nevertheless, the IC+GSK-J4/Emodin groups showed lower levels of all the inflammatory factors, compared with the IC group (Fig. 2b), suggested that IC-mediated inflammation was inhibited by emodin and GSK-J4.

**Figure 3:**
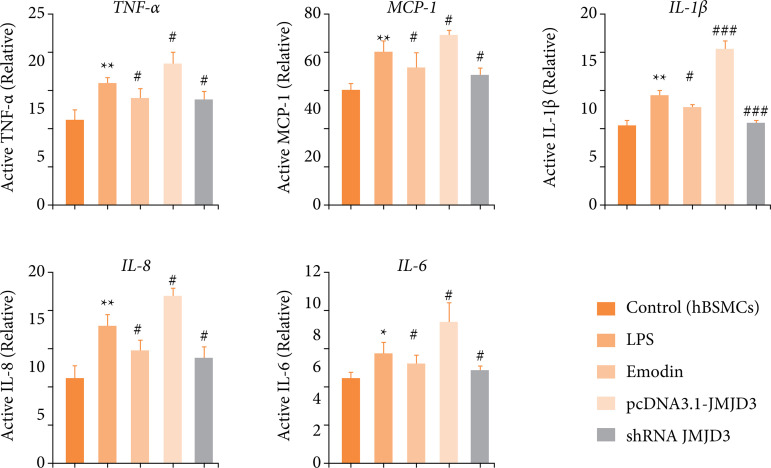
Emodin reduced the concentration of inflammatory substances *in-vitro* culture system with LPS. Expression levels of inflammation-related genes (TNF-α, MCP-1, IL-1β, IL-8 and IL-6) in hBSMCs was detected by enzyme-linked immunosorbent assay. Data are represented as the mean ± standard deviation.

Source: Elaborated by the authors. TNF: tumor necrosis factor; MCP: monocyte chemoattractant protein; IL: interleukin; LPS: lipopolysaccharide; hBSMC: human bladder smooth muscle cells; compared with control, * *P* < 0.05, ** *P* < 0.01; compared with IC group, **#** *P* < 0.05, **##** *P* < 0.01, **###** *P* < 0.001 represent significant differences between the indicated columns.

Consistently, to reveal the relationship between JMJD3 and inflammation, ELISA technology was used to detect intracellular expression levels of TNF-α, MCP-1, IL-1β, IL-8 and IL-6 in LPS-treated hBSMCs by transfecting JMJD3 plasmid and shRNA. The results showed that inflammatory conditions and JMJD3-overexpression significantly up-regulated the expression of TNF-α, MCP-1, IL-1β, IL-8 and IL-6 in hBSMCs; emodin or JMJD3-knockdown counter decreased these chemokines and cytokines (Fig. 3). Taken together, these results suggest emodin reduced IC severity and inflammatory pathology by regulating JMJD3.

### Emodin inhibits bladder fibrosis and extracellular matrix deposition in interstitial cystitis

**Figure 4:**
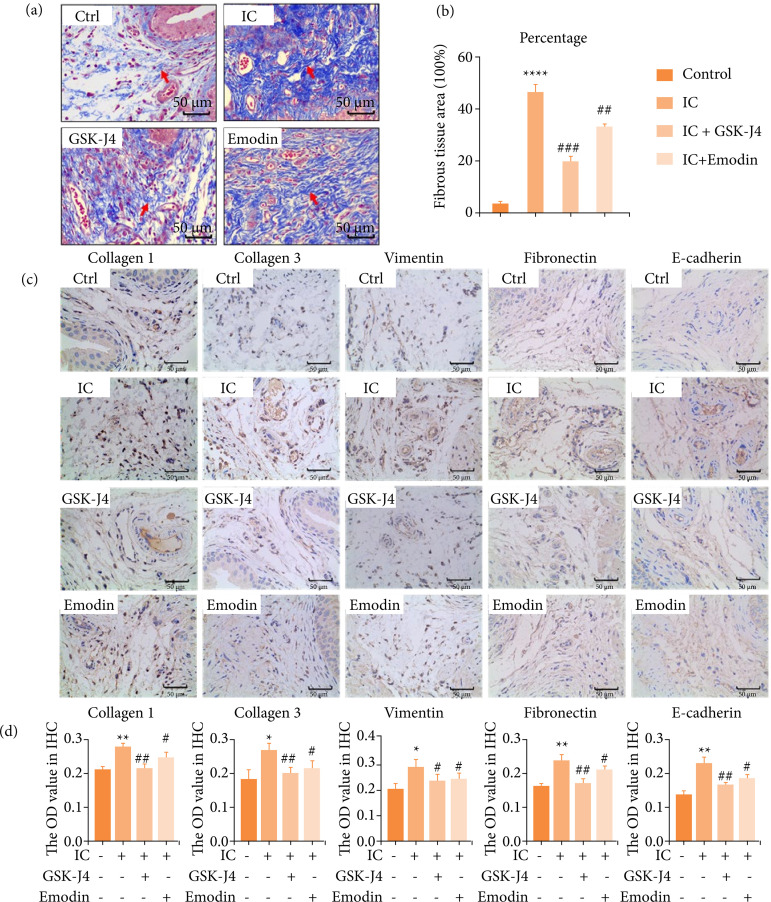
The degree and distribution of urinary bladder (UB) fibrosis in mice with cystitis after emodin treatment. **(a)** The extent of fibrosis in bladder interstitium was assessed with Masson staining. Scale bar = 50 μm final magnification. Red arrow: fibrous tissue (indigo dye part). **(b)** Quantitative analysis of the degree of tissue fibrosis in **(a)** (fibrotic tissue percentage). **(c)** Expression levels of fibrosis positive proteins assessed by immunohistochemistry. Scale bar = 50 μm final magnification. **(d)** Quantitative analysis of relative protein expression in **(c)** (high OD value represents high-protein expression). Mice that received CYP showed increased protein expression as compared to control mice, while mice that received CYP + emodin/or GSK-J4 showed reduced the expression. Data are represented as the mean ± standard deviation.

Source: Elaborated by the authors. Ctrl: control; IC: interstitial cystitis; OD: optical density; CYP: cyclophosphamide; compared with control, * *P* < 0.05, ** *P* < 0.01, **** *P* < 0.0001; compared with IC group, **#** *P* < 0.05, **##** *P* < 0.01, **###** *P* < 0.001 represent significant differences between the indicated columns.

Bladder fibrosis is an inevitable consequence of the development of interstitial cystitis^32^^,^ 33. Therefore, we evaluated the degree and distribution of bladder fibrosis. Consistently, compared with control, the blue collagen fibers in the IC group were significantly increased, and the bladder muscle was fibrotic, as evidenced by Masson staining. In mice treated with emodin and GSK-J4, the number of bladder collagen fibers decreased, and the fibrosis process was inhibited (Figs. 4a and 4b). Then, immunohistochemical analysis was performed for fibrosis-positive proteins and demonstrated that, after CYP treatment, collagen1, collagen3, vimentin, fibronectin levels increased in IC group relative to the control, whereas it decreased in the emodin and GSK-J4 groups compared to IC group (Figs. 4c and 4d). These results suggested that emodin and GSK-J4 could decrease the expression of fibrosis-related proteins, thus exerting an antifibrotic effect.

**Figure 5:**
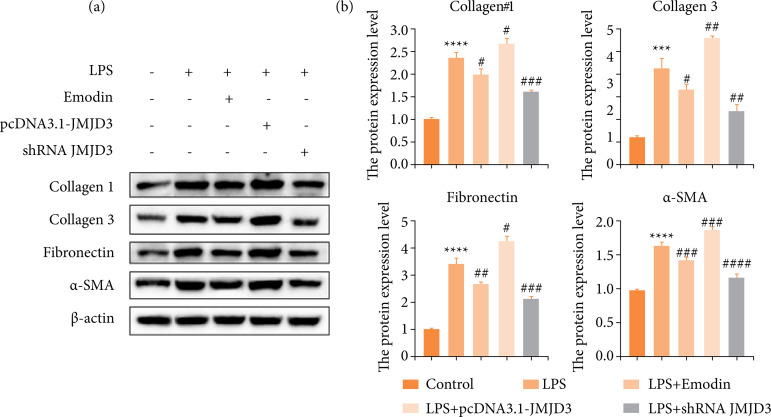
Emodin reduced the concentration of fibrosis-activated proteins *in-vitro* culture system with LPS. hBSMCs were pretreated with emodin, pcDNA3.1-JMJD3 and shRNA JMJD3 separately, followed by LPS for 24 hours. **(a)** Expression levels of fibrosis-positive proteins in LPS-induced inflammatory-hBSMCs assessed by Western blot. **(b)** Quantitative protein content. Data are represented as the mean ± standard deviation.

Source: Elaborated by the authors. LPS: lipopolysaccharide; hBSMC: human bladder smooth muscle cells; compared with control, ***P < 0.001, ****P < 0.0001; compared with LPS group, ^#^ *P* < 0.05, ^##^ *P* < 0.01, **###** *P* < 0.001, **####** *P* < 0.0001 represent significant differences between the indicated columns.

As before, we still verified at the cellular level. Excessive deposition of the ECM is one of the important causes of fibrotic phenotype^34^. We overexpressed or knocked down the JMJD3 protein and re-examined the expression of fibrotic proteins by Western blot in LPS-induced hBSMCs, including α-SMA, a recognized marker of myofibroblast activation. Our data discovered that LPS inducted-inflammatory environment and JMJD3-overexpression stimulated collagen1, collagen3, fibronectin and α-SMA significantly, promoting the development of fibrosis (Fig. 5). Consistently, emodin and JMJD3-knockdown down-regulated this abnormal elevation (Fig. 5). This indicates that high expression of JMJD lead to increased fibrosis, and emodin was able to reverse this damage. Combining our *in-vitro* and *in-viv*o results, we concluded that emodin could inhibit bladder fibrosis by weakening the expression of JMJD3.

### Emodin regulation JMJD3 via JAK/STAT, NF-κB and TGF-β/SMAD signal pathways

**Figure 6:**
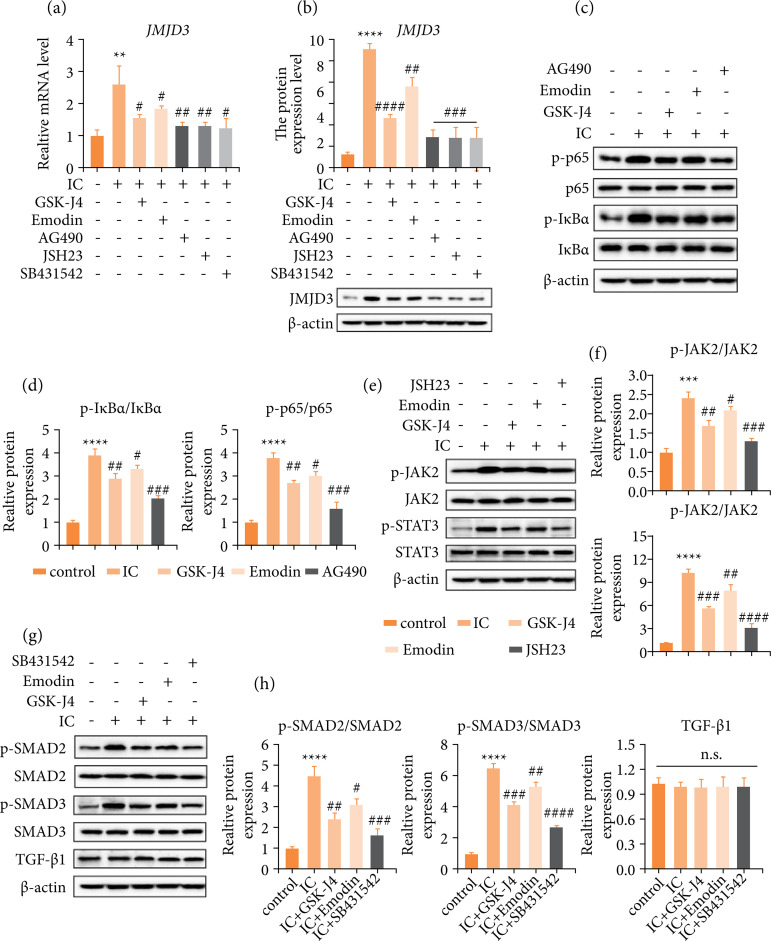
Emodin regulates JAK/STAT, NF-κB and TGF-β signal pathways to adjust the synthesis of JMJD3 and play a curative effect in interstitial cystitis. **(a)** Comparison of gene expression of JMJD3 among all groups by real-time quantitative polymerase chain reaction. **(b)** Both emodin and pathway inhibitors inhibited JMJD3 in IC-model mice detection and quantification by Western blot. **(c)** Inhibitor AG490 block NF-κB p65 and IκBα proteins phosphorylation activation, as does emodin. **(d)** Quantitative proteins content (including p-IκBα/IκBα and p-p65/p65). **(e)** Emodin and inhibitor JSH23 block JAK/STAT pathway factor JAK2 and STAT3 proteins phosphorylated. **(f)** Quantitative proteins content (including p-JAK2/JAK2 and p-STAT3/STAT3). **(g)** Emodin and inhibitor SB431542 block pathway proteins SMAD2 and SMAD3 phosphorylated. TGF-β1 expression was not affected by the drug. **(h)** Quantitative proteins content (including p-SMAD2/SMAD2, p-SMAD3/SMAD3 and TGF-β1). Data are represented as the mean ± standard deviation.

Source: Elaborated by the authors. IC: interstitial cystitis; TGF: tumor growth factor; N.s.: *P* ≥ 0.05, no statistical difference; compared with control, **P < 0.01, ***P < 0.001, ****P < 0.0001; compared with IC group, ^#^ *P* < 0.05, ^##^ *P* < 0.01, ^###^ *P* < 0.001, ^####^ *P* < 0.0001 represent significant differences between the indicated columns.

JMJD3 mediates inflammation. Published studies have reported that JAK/STAT, NF-κB and TGF-β/SMAD pathways participate in bladder interstitial inflammation pathogenesis, and JMJD3 was demonstrated to mediate those signals in different biological processes^35^^,^ 36. Therefore, we established a mouse model using CYP and administered specific inhibitors for blocking signal transduction in JAK/STAT, NF-κB, or TGF-β/SMAD pathways, investigated whether emodin functions through targeting these pathways in IC mice. Emodin, GSK-J4 and three pathway inhibitors all significantly inhibited JMJD3 transcription (Fig. 6a) and translation (Fig. 6b). Representative marker proteins in the selected pathways were evaluated by Western blot assay. Increased phosphorylation levels of pathways marker proteins as p65, IκBα (NF-κB; Figs. 6c and 6d), JAK2, STAT3 (JAK/STAT; Figs. 6e and 6f), SMAD2 and SMAD3 (TGF-β/SMAD; Figs. 6g and 6h) were observed in JMJD3-highly expressing IC mice.

An activation of JAK/STAT, NF-κB and TGF-β/SMAD signals was revealed, although the expression pattern of TGF-β did not appear to be altered (Figs. 6g and 6h). Then, the specific pathways inhibitors AG490, JSH23, SB431542 were utilized to confirm whether emodin functions through targeting JAK/STAT, NF-κB and TGF-β/SMAD signals. As shown in Figs. 6c–6h, AG490, JSH23 or SB431542 pretreatment rescued IC-induced inflammatory pathway activation, mainly by phosphorylation of inhibitory pathway proteins. Emodin had results in the same direction as these inhibitors, suggesting that emodin may also act as a pathway inhibitor, albeit to a lesser extent. The findings illustrate that the regulatory functions of emodin inhibits JMJD3 were achieved through targeting JAK/STAT, NF-κB and TGF-β/SMAD pathways.

## Discussion

IC/BPS is a severe disease that significantly reduces patients’ quality of life, and inflammation has emerged as a key constitutive element in the events cascade leading to IC^2^^,^ 5. For example, the levels of proinflammatory cytokines increase in IC patients^7^^,^ 37. Our study verified that the inflammatory pathology and fibrosis of IC were improved by emodin. Specifically, emodin reduces the amount of TNF-α, MCP-1, IL-1β, IL-8 and IL-6 in hBSMCs and tissues, protecting against inflammatory damage. As well as reducing the synthesis of collagen1, collagen3, vimentin, fibronectin and α-SMA, it inhibits the malignant development of fibrosis. Further research found JMJD3 overexpression has a direct response to the development of inflammation and fibrosis. Emodin inhibited the up-regulation of JMJD3 expression in CYP-induced cystitis mouse model and LPS-induced hBSMCs to delay the pathological progression of IC. We predicted the potential of emodin in the treatment of clinical interstitial cystitis.

Histone demethylase JMJD3 mediates multiple physiological and pathological processes, including cell differentiation, proliferation, autophagy, apoptosis and proinflammatory role^38^^,^ 39. According to reports, JMJD3 is regulated by JAK/STAT, NF-κB and TGF-β/SMAD signals^40^^–^ 42. We also demonstrated this, and that emodin regulates JMJD3 via JAK/STAT, NF-κB and TGF-β/SMAD. Interestingly, we found that administration of emodin prevented SMAD2 and SMAD3 phosphorylation, but it did not impair TGF-β1 expression (Figs. 6g and 6h). Transforming growth factor-β (TGF-β) is one of the main growth factors involved in driving endothelial-mesenchymal transition (EMT). TGF-β1 signals by activating TGF-β type I receptor (TβRI) and TGF-β type II receptor (TβRII) heteromeric complexes and activated TβRI phosphorylates receptor-associated SMAD2 and SMAD3^42^^,^ 43.

In our study, emodin does not appear to inhibit SMAD2 and SMAD3 phosphorylation by downregulating TGF-β1. Therefore, we speculate that emodin may regulate the TGF-β/SMAD pathway by other means, rather than changing the expression of TGF-β1. Furthermore, EMT appears to be stimulated primarily by the TGF-β2 isoform^44^^,^ 45, which could also signals through the SMAD pathway^46^. Emodin acting on TGF-β2 to regulate SMAD proteins phosphorylation may also be one of the reasons. These guesses proposed require follow-up attention to verification.

In recent years, the efficacy of various natural extracts or traditional Chinese medicine prescriptions is gradually being researched and revealed. Statistics show that more than 60% of the currently approved cancer drugs or drug candidates come from natural sources^47^^,^ 48. Among them, China ranked first in the number of disclosed patents for natural anticancer drugs, with 13,967, accounting for 68.35% of the global total^24^. Among them, emodin is widely used in clinical treatment, due to its wide range of pharmacological effects, such as anti-inflammatory, antibacterial, anti-tumor, hepatoprotective and laxative, etc.

In addition, emodin has also been reported to have certain protective effects on metabolic disorders and oxidative stress^49^. In a new study, researchers from the School of Medicine of University of South Carolina found that emodin prevents colon cancer in mice by the ability to reduce the number of M2-like macrophages^50^. The precise regulation of macrophages polarization is very important to maintain the health of the body^51^. Researchers pointed that changing the metabolism of macrophages may protect the body from overload, while also addressing the body’s inflammatory response^52^. However, whether emodin regulates the body’s inflammatory response (and thus fibrosis) by targeting macrophages, and its molecular mechanism remains to be further investigated.

In conclusion, we report here that emodin and GSK-J4 effectively eased the severity of IC by impairing the transcription and translation of the JMJD3 via the JAK/STAT, NF-κB and TGF-β/SMAD pathways, thereby decreasing levels of systemic cytokines. Our study supports the efficacy of emodin as a potential treatment for IC and provides an opportunity for developing novel therapeutic agents for clinical IC. We must admit that the therapeutic effect of a single emodin is still not as good as that of the JMJD3 specific blocker GSK-J4. However, emodin inhibits the proliferation of various cancers and can effectively combine with other treatments. Perhaps emodin combined with other drugs in the treatment of IC will become a direction of future exploration.

## Conclusion

Our results suggested that emodin impaired the transcription and translation of the JMJD3 via the JAK/STAT, NF-κB and TGF-β/SMAD signaling pathways, thereby decreasing levels of systemic inflammatory cytokines and fibrosis markers, and thus effectively alleviating the severity of IC. Our study supports the efficacy of emodin as a potential treatment for IC and other inflammatory diseases.

## Data Availability

The data will be available upon request.
